# Combining Statins with Radiotherapy for Prostate Cancer: From Photon Experience to Proton Potential

**DOI:** 10.3390/jcm15020568

**Published:** 2026-01-10

**Authors:** Mohammad Saki, Mark E. Artz, Jiyeon Park, Perry B. Johnson, Curtis Bryant, K. C. Balaji, Hardev Grewal

**Affiliations:** 1 Department of Radiation Oncology, University of Florida College of Medicine, Gainesville, FL 32610, USA; msaki@ufl.edu (M.S.);; 2 University of Florida Health Proton Therapy, Jacksonville, FL 32206, USA; 3 Southwest Florida Proton, Fort Myers, FL 33967, USA; 4 Department of Urology, University of Florida College of Medicine, Jacksonville, FL 32209, USA

**Keywords:** prostate cancer, proton therapy, statins, radiosensitization, clinical trials

## Abstract

Statins have shown promise as radiosensitizers in photon-based radiotherapy (RT), with studies demonstrating improved biochemical recurrence-free survival and reduced toxicity in prostate and other solid tumors. However, existing data derived entirely from photon-based RT and the potential synergy with proton therapy remain hypothetical at this stage. The current narrative review extrapolates the therapeutic benefits of statins observed in photon-based RT to proton therapy (PBT) to enhance therapeutic efficacy. The proposed combination of statins and PBT is a theoretical extension grounded in the mechanistic overlap between statin-induced radiosensitization and proton-specific advantages in dose conformity and linear energy transfer (LET). The hypothesis of enhanced synergy between statins and PBT warrants systematic preclinical testing and clinical trials before translation into standard practice.

## 1. Introduction

Prostate cancer is projected to remain the leading cancer among men in the United States, accounting for 30% of all cancer diagnoses in men and ranking as the second leading cause of cancer-related death [1]. An estimated 313,780 new cases and 35,770 deaths are expected in 2025 [2]. Advances in radiation therapy have significantly improved treatment outcomes in patients with prostate cancer. Proton beam therapy (PBT) has demonstrated rapid clinical implementation, particularly in the management of prostate cancer. However, a major knowledge gap exists within both preclinical and clinical investigations regarding the concurrent administration of PBT with small-molecule drugs. While debates persist regarding the comparative benefits of intensity modulated radiotherapy (IMRT) versus PBT in minimizing radiation-induced toxicity, PBT demonstrates distinct advantages through its precise dose deposition and higher relative biological effectiveness (RBE), offering compelling potential to further reduce toxicity and enhance therapeutic efficacy. These physical and biological properties are discussed in detail in the results and discussion section to avoid redundancy.

Statins, inhibitors of 3-hydroxy-3-methylglutaryl-coenzyme A (HMG-CoA) reductase, are widely prescribed for dyslipidemia and cardiovascular disease prevention. Beyond lipid regulation, statins exhibit pleiotropic effects such as anti-inflammatory properties, modulation of the tumor microenvironment, and radiosensitization [3,4]. Emerging observational studies in oncology suggest better outcomes for statin users, underscoring the need for clinical trials to investigate their therapeutic potential [5] and their systemic radioprotective benefits in prostate cancer PBT.

The objective of this work is to explore the potential for clinical benefit when statin use is combined with radiotherapy and the mechanisms that likely contribute to this effect, emphasizing photon-derived evidence while outlining theoretical implications for proton therapy. Despite encouraging findings, evidence for statin-mediated radiosensitization remains heterogeneous, influenced by statin type (lipophilic vs. hydrophilic), dose, and patient comorbidities. The current work synthesizes evidence across preclinical, retrospective, and prospective levels and explores the hypothesis that combining statins with PBT could further optimize the therapeutic ratio.

## 2. Materials and Methods

### 2.1. Literature Search Strategy (PubMed 2000–2025)

This study was designed as a narrative review rather than a systematic review to identify relevant studies examining the combination of statins with radiotherapy in cancer treatment, with particular emphasis on prostate cancer. The search was performed using the PubMed database from 2000 through June 2025. The following search terms were used in various combinations: statin, atorvastatin, simvastatin, lovastatin, pravastatin, or rosuvastatin combined with radiotherapy, radiation therapy, proton therapy, photon therapy, or brachytherapy, and further combined with prostate cancer or prostatic neoplasms. Additional searches included radiosensitization or radioprotection combined with statin.

### 2.2. Inclusion and Exclusion Criteria

Studies were included if they examined statins in combination with any form of radiotherapy, preclinical studies investigating statin mechanisms relevant to radiation, clinical studies reporting outcomes of statin use during radiotherapy, studies in prostate cancer or other solid tumors with mechanisms applicable to prostate cancer, studies examining oncologic effects of statins in cancer to identify mechanisms potentially involved in radiosensitization, and articles published in English. Inclusion criteria encompassed in vitro, in vivo, retrospective, meta-analytic, and prospective human studies evaluating the interaction between statins and radiation response.

Studies were excluded if they combined statins with cancer treatment modalities other than radiotherapy, such as chemotherapy alone or surgery alone, focused on hematologic malignancies only, were irrelevant review articles without original data, case reports, and letters to the editor, or examined statin effects unrelated to cancer treatment.

Two reviewers independently screened titles and abstracts, followed by a full-text review of potentially relevant articles. Data extracted included study design, patient/model characteristics, statin type and dosing, radiation parameters, endpoints, and quantitative results where available.

AI tools (ChatGPT-4 (OpenAI) and Claude Sonnet 4.5 (Anthropic)) were used for language editing and table formatting. All content was reviewed and verified by the authors for accuracy.

### 2.3. Categorization by Evidence Level

Given the narrative nature of this review and the heterogeneity of study designs, formal quality assessment tools were not uniformly applied. However, studies were categorized by evidence level (Level 1: in vitro, Level 2: in vivo, Level 3: retrospective clinical, Level 4: meta-analyses, Level 5: prospective trials) to provide transparency regarding the strength of evidence.

#### Limitations

This narrative review was not conducted according to systematic review guidelines (PRISMA). The approach was chosen due to the heterogeneous nature of available evidence and the exploratory nature of the statin–proton therapy hypothesis. A comprehensive systematic review and meta-analysis may be warranted as more standardized evidence emerges.

## 3. Results

Radiation therapy is integral to the management of localized and advanced prostate cancer. Despite significant advancements in EBRT techniques and technology, radiation-induced gastrointestinal (GI) and genitourinary (GU) toxicities remain challenges, particularly in patients with diabetes and at the higher doses required for optimal tumor control [6]. PBT addresses these limitations by minimizing the integral dose, reducing late toxicities, and lowering secondary cancer risks [7].

Recent comparative studies have shown mixed results regarding proton therapy’s clinical advantages over photon-based treatments. Some studies demonstrate reduced urinary toxicity but increased bowel toxicity [8], while others show no significant differences in toxicity profiles [9,10]. Current comparative effectiveness studies may be limited by study design, follow-up duration, and the lack of synergistic approaches. Nonetheless, lower risk of secondary cancers [11] as well as the consistent dosimetric advantages of proton therapy suggest unrealized potential that may be enhanced through innovative combination strategies such as concurrent statin therapy.

The biologically equivalent dose (BED) studies demonstrate that dose escalation improves biochemical control, with higher BED correlated with better freedom from biochemical failure (FFBF) in prostate cancer patients [12]. This principle is demonstrated in the accelerated hypofractionated proton therapy study, which achieved excellent 5- and 7-year outcomes (96.8%/95.2% freedom from biochemical progression) with minimal GI/GU toxicity [13], suggesting that proton therapy’s superior dose distribution enables effective dose escalation with acceptable toxicity profiles [13,14].

When comparing 5-year FFBF in high-risk prostate cancer patients treated with similar physical BED, PBT has been reported to have up to 20% higher 5-year FFBF than photon therapy; at BED of 157 Gy, photon therapy yields a predicted five-year FFBF of 50%, while proton therapy achieves 73% [15,16]. Although it has been reported that there are no statistically significant differences in GI or GU toxicity between PBT and IMRT at 6-, 12-, and 24 months post-treatment [9], long-term follow-up studies (>5 years) are essential to fully evaluate late effects and establish definitive comparative effectiveness.

Both proton and photon therapy are effective in treating prostate cancer. However, debates persist regarding the cost-effectiveness of proton therapy and its marginal benefits in certain patient subsets [17]. Nonetheless, the reduction in the integral dose during proton therapy remains a distinct advantage, particularly when paired with statins to enhance therapeutic outcomes. Additionally, the higher RBE of protons compared to photons may further enhance their therapeutic efficacy, especially in targeting radioresistant tumor cells [18,19].

Statins function primarily by inhibiting 3-hydroxy-3-methylglutaryl coenzyme A (HMG-CoA) reductase, a key enzyme in the cholesterol biosynthesis pathway. Through this primary mechanism and subsequent downstream events, statins enhance radiosensitivity across multiple complementary pathways that synergistically enhance tumor cell killing while protecting normal tissues.

DNA repair and survival pathways: Korte et al. demonstrated that ionizing radiation significantly reduces the proliferation of prostate cancer cell lines, with a synergistic effect observed when combined with simvastatin, likely due to the inhibition of HMG-CoA reductase [20]. In this context, studies have shown that pitavastatin, a lipophilic form of statin, increases persistent DNA double-strand breaks, induces cellular senescence, and enhances radiation effects on radioresistant tumors through inhibition of protein prenylation via HMG-CoA reductase, farnesyl diphosphate synthase, and protein farnesyl transferase [21]. Inhibition of HMG-CoA reductase blocks the mevalonate pathway, a critical biosynthetic route for cholesterol and isoprenoid synthesis, and prevents prenylation of small GTPases (Ras, Rho), which disrupts pro-survival signaling events via the Ras/Raf/MEK and the PI3K/AKT/mTOR pathway [22,23,24,25]. Statins also compromise homologous recombination repair by downregulating RAD51 expression, thereby impairing DNA double-strand break repair machinery [26,27], which yields prolonged persistence of radiation-induced DNA damage and enhanced therapeutic efficacy.

Cell cycle arrest and apoptosis: Statins reduce prostate cancer cell viability by inducing apoptosis and G1 cell cycle arrest upon caspase activation, suppression of cell cycle regulators, and RhoA inactivation [28,29]. Apoptosis via autophagy activation has also been shown to mediate the therapeutic effects of atorvastatin in prostate cancer cells [30], with mechanistic studies revealing that statin treatment modulates 22 of 86 autophagy-related genes [31]. When key autophagy mediators Atg7 and Atg12 were knocked down using siRNA, both radiosensitivity and apoptosis induction were substantially diminished, confirming the critical role of autophagy in statins’ therapeutic effects [31].

Tumor hypoxia and HIF-1α: Statins reduce hypoxia, a major factor limiting radiotherapy efficacy, through downregulation of hypoxia-inducible factor 1-alpha (HIF-1α) and enhancement of nitric oxide production [32,33,34,35]. HIF-1α is a key transcription factor that mediates cellular adaptation to low oxygen environments and promotes radioresistance. By inhibiting HIF-1α, statins reduce the hypoxic fraction of tumors and enhance their sensitivity to radiation therapy. Additionally, statins have been shown to enhance nitric oxide synthase activity, leading to increased nitric oxide (NO) production, as comprehensively reviewed by Gorabi et al. [36]. NO acts as a potent vasodilator that relaxes vascular smooth muscle and increases blood flow, thereby improving oxygen delivery to hypoxic tissues, including tumors, which enhances radiosensitivity [37].

Immune modulation and anti-inflammatory effects: Complementary studies demonstrate that statins have dose-dependent immunomodulatory effects on regulatory T cells (Tregs) across multiple diseases. At therapeutic concentrations, statins enhance Treg numbers, function, and tissue-specific redistribution in atherosclerosis, rheumatoid arthritis, and stroke, providing beneficial anti-inflammatory effects [38,39,40]. However, in vitro mechanistic studies reveal that higher atorvastatin concentrations (10 μM) paradoxically impair Treg suppressive capacity [41], indicating concentration-dependent biphasic effects on immune regulation. In cancer therapy, statin-enhanced Treg function may compromise antitumor immunity by promoting immune evasion, requiring careful balance between the radiosensitizing benefits and potential immunosuppressive effects during radiotherapy. However, enhanced Treg function may still be beneficial by reducing radiation-induced inflammation and protecting normal tissues from treatment-related toxicity, potentially improving the therapeutic window between tumor control and normal tissue damage.

Statins reduce interleukin-6 (IL-6) and tumor necrosis factor-alpha (TNF-α) levels, inhibit NF-κB signaling, and mitigate radiation-induced inflammation and fibrosis. Lovastatin inhibits TNF-α-induced E-selectin expression in primary human endothelial cells through impaired protein geranylgeranylation and Rho signaling disruption, which may offer clinical benefits in preventing E-selectin-mediated metastasis [42]. It has been shown that lovastatin provides radioprotective effects in normal tissues of healthy mice by decreasing NF-κB activation and inflammatory markers (TNFα, IL-6), and reducing fibrotic gene expression (TGFβ, CTGF, collagen) [43]. Importantly, lovastatin did not affect DNA damage induction (γH2AX phosphorylation) in normal tissues [43]. This suggests statins may selectively protect healthy tissues from radiotherapy toxicity without compromising the prolonged DNA damage effects that enhance antitumor efficacy.

Since radiation therapy often triggers inflammatory responses that lead to normal tissue damage and fibrosis, this dual effect of protecting normal tissues while enhancing tumor radiosensitivity makes statins particularly promising adjuncts to radiotherapy. The anti-inflammatory properties of statins may reduce both acute and late radiation toxicities while preserving or enhancing antitumor efficacy.

### 3.1. Preclinical Evidence, Level 1 In Vitro and Level 2 In Vivo Studies

**Figure 1:**
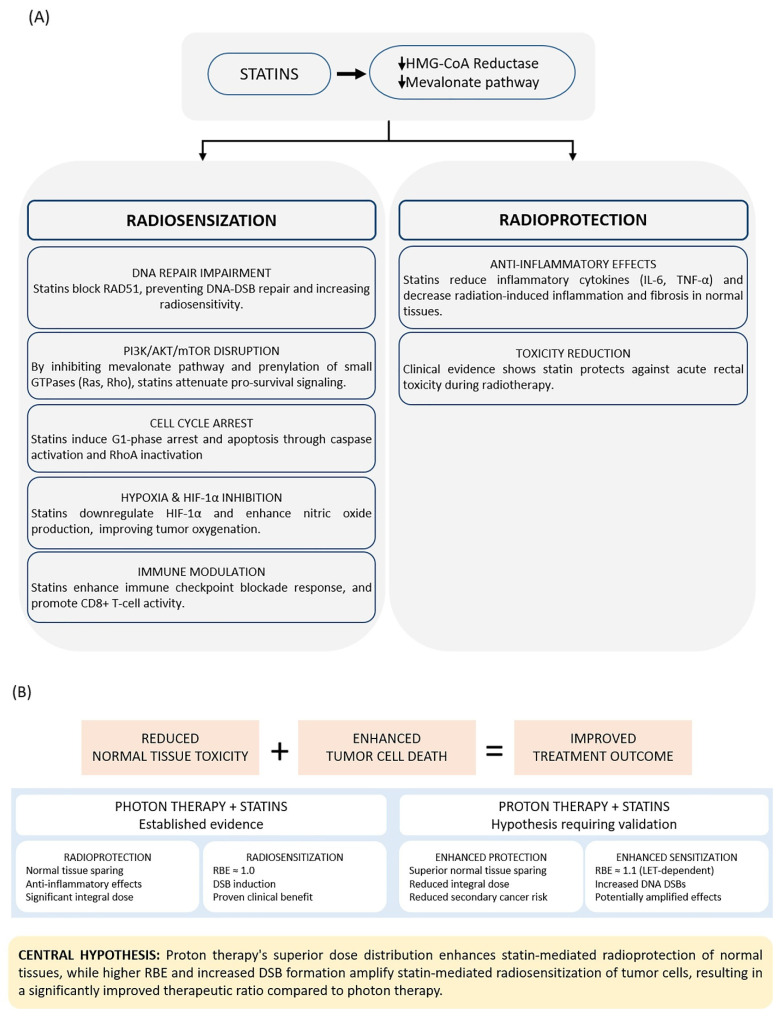
(**A**) Statins exert tissue-selective dual effects through HMG-CoA reductase inhibition. Radiosensitization mechanisms primarily target tumor cells through DNA repair inhibition, PI3K/AKT/mTOR pathway disruption, cell cycle arrest, hypoxia mitigation, and immune modulation. Radioprotection mechanisms primarily benefit normal tissues through cytokine suppression, E-selectin inhibition, and clinical toxicity reduction. (**B**) Proton therapy may enhance both protective and sensitizing effects compared to photon therapy through superior dose distribution and higher relative biological effectiveness. Abbreviations: DSB, double-strand break; HIF-1α, hypoxia-inducible factor 1-alpha; IL-6, interleukin-6; LET, linear energy transfer; RBE, relative biological effectiveness; TNF-α, tumor necrosis factor-alpha; Treg, regulatory T cell. Downward arrows (↓) indicate decreased activity or expression.

The mechanisms by which statins enhance radiotherapy efficacy while minimizing adverse effects are illustrated and summarized in Figure 1. Multiple in vitro and in vivo studies have demonstrated enhanced radiosensitivity and radiotherapy efficacy when statins are combined with photon irradiation in cancer cell lines and animal models (Table 1 and Table 2). Brown et al. demonstrated that lipophilic statins suppress prostate cancer cell migration and colony formation in human bone marrow stroma by inhibiting geranylgeranyl pyrophosphate production, thereby reducing the dissemination of metastatic cells [44]. Notably, pravastatin, a hydrophilic type of statin, showed no significant effect on invasion, suggesting that lipophilic statins may be more effective for combination therapy [44].

Yu et al. reported that atorvastatin enhanced the cytotoxic effects of photon irradiation in prostate cancer cells by reducing endogenous reactive oxygen species (ROS) levels and increasing the persistence of radiation-induced ROS [48]. Similarly, laboratory and animal studies have demonstrated that treatment with human chorionic gonadotropin (hCG) combined with lovastatin and radiation of PC-3 flank tumors significantly reduced tumor volume, decreased ex vivo colony survival, and increased pro-caspase 3 cleavage while reducing Ki67 levels [46,47].

Wang et al. performed a zebrafish chemical genetic screen that identified rosuvastatin as having significant antiangiogenic and antitumor activities, demonstrating suppression of xenografted PPC-1 prostate tumors in NOD-SCID mice with decreased microvessel density and tumor cell apoptosis [53]. In vivo studies have confirmed radioprotective effects in normal tissues. Nuble et al. reported that lovastatin protects primary human umbilical vein endothelial cells (HUVECs) from EBRT-induced cell death without conferring radioresistance to human fibroblasts [54]. Preclinical studies indicate that statins mitigate photon EBRT-induced fibrosis and inflammation in normal tissues such as intestines [51] and suppress acute and subchronic pro-inflammatory and pro-fibrotic responses [43].

Collectively, in vitro and in vivo studies demonstrate consistent radiosensitization with lipophilic statins through DNA repair inhibition, apoptosis induction, hypoxia modulation, and suppression of pro-survival signaling. Pravastatin shows limited efficacy, highlighting the importance of lipophilicity. Evidence of normal-tissue radioprotection further strengthens the rationale for therapeutic exploration.

### 3.2. Retrospective Clinical Evidence in Prostate Cancer, Level 3

Several large retrospective cohorts have evaluated statin use during definitive photon RT for prostate cancer. Most demonstrate improved biochemical recurrence-free survival (bRFS) among concurrent users (Table 3). Li et al. [55] conducted a population-based cohort study in Taiwan of 567 prostate cancer patients with hyperlipidemia who received radiotherapy. Patients who used statins after prostate cancer diagnosis had significantly longer average survival times (9.3 years) and were associated with a lower risk of mortality (aHR = 0.24, 95% CI = 0.09–0.66) compared to patients who did not use statins. Kaulanjan et al. [56] evaluated 3555 patients undergoing different radiation therapy modalities and found that statin use was not universally beneficial across all radiation techniques, highlighting the importance of treatment modality considerations. Murtola et al. [57] studied 6537 prostate cancer cases from the Finnish randomized study of screening for prostate cancer and found that statin use after diagnosis was associated with decreased risk of prostate cancer death (HR 0.80; 95% CI 0.65–0.98), with the risk decrease being clearest in men managed with androgen deprivation therapy.

Kollmeier et al. demonstrated improved biochemical outcomes with statin use in patients with high-risk localized prostate cancer treated with radiotherapy, showing a hazard ratio of 0.52 (*p* = 0.02) for PSA relapse-free survival in high-risk patients, compared to no significant improvement in intermediate or low-risk groups [58]. Gutt et al. reported similar findings across all risk groups, with hazard ratios of 0.43 (*p* < 0.001) for freedom from biochemical failure [59]. Oh et al. [60] found that among 247 men treated with permanent Iodine-125 brachytherapy, statin use was associated with significantly improved freedom from biochemical failure (HR 0.28; 95% CI 0.10–0.72; *p* < 0.01) and remained significant in multivariate analysis (HR 0.288; 95% CI 0.086–0.886; *p* = 0.0299). Park et al. showed that statins may provide therapeutic benefits in prostate cancer patients treated with RT, while no such effect was observed in those undergoing radical prostatectomy, emphasizing the importance of considering primary treatment modalities when evaluating the impact of statins on oncologic outcomes [62].

Retrospective photon RT data suggest a consistent signal of improved biochemical control and survival in statin users, with variability linked to study design and patient selection. Across studies, statin benefit appears strongest in high-risk or post-diagnosis cohorts; however, heterogeneity in dose, duration, and RT technique limits cross-comparison, and several analyses report neutral findings. In addition, none of these studies specifically involved proton therapy; all were based on photon modalities such as 3D-CRT, IMRT, or VMAT.

### 3.3. Comprehensive Meta-Analysis Evidence: Level 4

Combined meta-analytic evidence indicates that statin use during or after radiotherapy correlates with lower recurrence and improved overall survival, particularly in high-risk prostate cancer. Sun et al. [63] conducted a systematic review and meta-analysis of 33 cohort studies, finding that statin use was significantly associated with a 14% reduction in the hazard ratio of biochemical recurrence (pHR: 0.86, 95% CI: 0.78–0.95) and a 26% reduction in the risk ratio of biochemical recurrence (pRR: 0.74, 95% CI: 0.57–0.94). Importantly, subgroup analysis showed that statins could result in a 22% reduction in the hazard ratio of biochemical recurrence among patients accepting radiotherapy (pHR: 0.78, 95% CI: 0.61–0.98). An et al. [64] analyzed 24 cohort studies involving 369,206 participants and found that statin use significantly reduced the risk of prostate cancer-specific mortality with a pooled hazard ratio of 0.76 (95% CI: 0.69–0.84), especially for post-diagnostic statin users (pHR = 0.81, 95% CI: 0.77–0.85) and patients who received radiotherapy (pHR = 0.68, 95% CI: 0.50–0.93). Another meta-analysis of 27 studies performed by Yin et al. [65] demonstrated that statin use was associated with a potential tendency to improve biochemical recurrence-free survival in patients undergoing curative treatment (*p* = 0.05), with particular benefit in high-risk patients (*p* < 0.01). These results are summarized in Table 4.

Meta-analyses provide the strongest pooled evidence of benefit, demonstrating reduced recurrence and mortality among radiotherapy patients who use statins, particularly post-diagnostic users. However, outcome variability and differences in radiation modalities and statin regimens remain notable limitations.

### 3.4. Ongoing Prospective Trials, Level 5

To date, no dedicated prospective trials have evaluated statin and RT synergy in prostate cancer. The only ongoing randomized controlled trial (Patil et al. CTRI/2018/11/016459) investigates rosuvastatin (20 mg daily) combined with neoadjuvant chemoradiation in 316 patients with locally advanced rectal cancer [68]. If positive, this trial will provide Level 1 evidence for statin–radiation combinations and may establish a foundation for similar investigations in prostate cancer patients undergoing radiotherapy, including proton beam therapy (Table 5). Overall, Phase II evidence suggests feasibility and potential toxicity mitigation; however, these data cannot be extrapolated directly to prostate cancer or to proton therapy. Absence of prostate-specific prospective studies constitutes a major knowledge gap.

### 3.5. Toxicity Considerations and Negative Results

Several retrospective analyses have proposed that statins may protect normal tissues from radiation-induced injury. Palumbo et al. [70] conducted a prospective observational study of 195 patients receiving hypofractionated IMRT and found that statin use significantly reduced both the incidence and grade of acute rectal toxicity in multivariate analysis. Anscher et al. [69] conducted a Phase II study specifically designed to prevent radiation-induced rectal injury with lovastatin. The study found that 38% of patients developed grade 2 or higher toxicity during 2-year follow-up, indicating that the primary endpoint was not met (Table 5). Interestingly, lovastatin did not reduce the incidence of grade 2 or higher rectal toxicity compared with historical controls. This negative result with photon therapy may present an opportunity for proton therapy investigation, as proton therapy’s superior dose distribution significantly reduces rectal dose exposure.

The results from Chao et al. [61] did not find a benefit for statin therapy in prostate cancer recurrence among patients who underwent radiation therapy in a cohort of 774 patients. Similarly, Soto et al. [71] reported no effect of hydrophobic or hydrophilic statins on biochemical outcomes after radiotherapy for localized prostate cancer in 968 patients.

Cuaron et al. [72] found no association between statin use and improved outcomes in intermediate- and high-risk patients treated with brachytherapy. Scosyrev et al. [67] conducted a meta-analysis of eight cohort studies that did not definitively support the hypothesis that statins influence the risk of biochemical recurrence, though considerable heterogeneity was observed between studies.

Taken together, potential toxicity reduction with statins is promising yet remains inconsistent with possible benefits in vascular and rectal endpoints. Therefore, the dual behavior (tumor radiosensitization and normal-tissue protection) supports further investigation into differential biological responses to statins.

### 3.6. Evidence from Other Cancer Types

Emerging clinical data demonstrate significant therapeutic benefits of statin use across multiple cancer types treated with conventional photon-based radiotherapy (Table 6). Statin therapy improves survival in patients with unresectable stage III lung squamous cell carcinoma (LSCC) undergoing concurrent chemoradiotherapy, with a dose–response relationship indicating that higher statin utilization reduces lung cancer-specific mortality [73]. Studies in esophageal squamous cell carcinoma (ESCC) indicate improved overall survival and reduced therapy-related complications with statin use during concurrent chemoradiotherapy [74]. Furthermore, a meta-analysis of 35 trials reported that statins decrease the hazard ratio for cancer-specific mortality in urologic cancers [66].

## 4. Discussion

### 4.1. Strength of Evidence and Limitations

The evidence for combining statins with radiation therapy in prostate cancer spans multiple levels, from mechanistic studies to retrospective clinical investigations and comprehensive meta-analyses, with increasingly consistent findings suggesting clinical benefit. The largest meta-analysis by An et al. involving 369,206 patients across 24 cohort studies provides robust evidence supporting statin use in radiotherapy patients, particularly for high-risk disease [64]. However, several important limitations must be acknowledged when evaluating the potential synergistic effects of statins with radiation therapy. Despite encouraging mechanistic and clinical signals, the available literature contains considerable limitations. Retrospective analyses are subject to selection bias, confounding cardiovascular comorbidities, inconsistent statin dosing, and variable treatment duration. Radiotherapy parameters, including technique, dose, and fractionation, differ substantially across studies, limiting direct comparability. Furthermore, mechanistic studies utilize different statin types, concentrations, and experimental environments, making uniform conclusions challenging. These limitations underscore the need for controlled preclinical and prospective clinical studies in proton therapy settings.

While recent meta-analyses show overall benefit, individual studies demonstrate considerable inconsistency in outcomes, with some high-quality studies showing no benefit or even potential harm in specific populations (Table 7). Most importantly, no published studies have directly examined statin–proton therapy combinations, making all conclusions regarding PBT synergy speculative and hypothesis-generating rather than evidence-based.

### 4.2. Optimal Statin Choice and Patient Selection

Based on the evidence presented, lipophilic statins appear to be the optimal choice for combination with radiotherapy. The in vitro studies clearly demonstrate that lipophilic statins (atorvastatin, simvastatin, and lovastatin) consistently enhance radiosensitivity across multiple prostate cancer cell lines, while pravastatin, a hydrophilic statin, showed no significant effect on cancer cell invasion [44]. This superior efficacy of lipophilic statins likely stems from their enhanced cellular penetration and ability to cross cell membranes more effectively than their hydrophilic counterparts. Among the lipophilic options, atorvastatin and simvastatin showed particularly robust effects in both hypoxic conditions and DNA repair inhibition, suggesting these may be preferred agents. Moyad et al. [81] found that atorvastatin showed superior biochemical progression-free survival outcomes compared to other statins in brachytherapy patients, with 97.8% of patients taking atorvastatin compared to 94.7% taking other statins being free of biochemical progression.

Clinical dosing guidance can be informed by the Mace et al. rectal cancer study [76], which demonstrated significant pathologic response improvement using atorvastatin at a mean dose of 31.3 mg/day and simvastatin at 27.8 mg/day during neoadjuvant chemoradiation. Patient stratification represents a critical consideration for optimizing statin–radiotherapy combinations. High-risk prostate cancer patients consistently demonstrate the most significant benefit across multiple studies, with hazard ratios ranging from 0.52 to 0.78 for biochemical recurrence-free survival compared to minimal or no benefit in low- and intermediate-risk groups. Li et al.’s study specifically focused on patients with hyperlipidemia, suggesting that patients with pre-existing cardiovascular indications for statins represent an ideal target population for dual therapeutic benefit [55]. The specificity of benefit to biochemical recurrence rather than distant metastasis-free survival indicates that statins primarily enhance local–regional control, making patients with locally advanced disease particularly suitable candidates. Hamilton et al. [77] demonstrated that in men treated with androgen deprivation therapy following radiotherapy, statin use was associated with improved overall survival (HR: 0.64; 95% CI 0.53–0.78) and prostate cancer-specific survival (HR: 0.65, 95% CI 0.48–0.87), suggesting particular benefit in this population. Future patient selection strategies should incorporate predictive biomarkers, baseline cardiovascular risk profiles, and tumor characteristics such as hypoxic status to further refine this promising combination approach.

### 4.3. Timing and Duration

The timing of statin administration relative to radiation delivery, duration of treatment, and potential need for dose modifications based on individual patient factors all require further investigation to maximize therapeutic benefit while minimizing potential adverse effects. Current evidence suggests that concurrent administration during radiotherapy is most beneficial, but optimal pre-treatment initiation timing and post-treatment continuation duration remain undefined. Murtola et al. found that post-diagnostic statin use was associated with decreased mortality risk while pre-diagnostic use was not associated with benefit [57], suggesting that timing of initiation relative to cancer diagnosis and treatment remains critical.

Immediate research priorities should include direct evaluation of statin effects in proton radiation environments, LET-dependent radiosensitization studies, RBE modulation assessments with various statin types, and optimization of statin dosing and timing relative to proton delivery. Well-designed prospective clinical trials are essential to definitively establish the safety, efficacy, and optimal protocols for statin–radiotherapy combinations. These trials should incorporate standardized statin dosing and administration protocols, biomarker-driven patient selection, comprehensive toxicity assessments, and long-term follow-up for late effects evaluation. The ongoing Patil et al. randomized controlled trial in rectal cancer may provide important insights that could inform future prostate cancer studies, particularly regarding optimal statin selection and dosing strategies.

### 4.4. Hypothesis for Proton and Statin Synergy

The mechanistic evidence presented suggests that statin–proton therapy combinations may theoretically offer superior outcomes compared to statin–photon combinations, though this hypothesis requires critical examination. The enhanced DNA damage hypothesis rests on the assumption that proton therapy’s increased DSB induction would synergize with statin-mediated repair inhibition. However, this presumes that the temporal dynamics of statin effects align precisely with the proton damage kinetics, an assumption not yet demonstrated experimentally. The degree to which statins actually impair DNA repair machinery specifically during the critical post-irradiation window remains unclear, and whether this impairment is sufficient to meaningfully augment proton-induced damage represents a testable but unproven proposition.

Limited data from proton therapy cohorts provide insufficient evidence to support assertions of reduced late toxicity when statins are added to treatment regimens. Statins may reduce inflammation and vascular injury that contribute to radiation-induced tissue damage, yet whether this anti-inflammatory effect translates to genuinely less structural damage or simply delays the clinical manifestation of toxicity remains unclear. Until prospective studies with adequate follow-up periods systematically evaluate late effects in patients receiving combined statin–proton therapy, claims of reduced complications remain speculative projections rather than evidence-based conclusions.

The improved therapeutic ratio argument depends on proton therapy’s superior normal tissue sparing, allowing for more aggressive statin dosing or radiotherapy doses. Yet, this advantage exists only if statins genuinely enhance tumor control without proportionally increasing normal tissue toxicity, a differential effect that cannot be assumed. The clinical reality of proton therapy involves prescription methodology differences from photon IMRT: proton prescriptions typically employ CTV-robust optimization accounting for range uncertainty, whereas IMRT prescribes to PTV coverage thresholds exceeding 95%. This methodological distinction, combined with proton therapy’s requirement for extensive daily image guidance due to anatomical sensitivity and longer treatment delivery times for beam-specific QA and range verification, introduces practical constraints. These operational complexities increase treatment cost and duration, raising the question of whether marginal biological gains from statin synergy justify these resource investments.

The hypoxia consideration requires particular scrutiny regarding fundamental radiobiological principles. Hypoxia sensitization represents a well-established phenomenon for photon radiation, which operates primarily through indirect ionization mechanisms requiring oxygen to fix DNA damage via free radical intermediates. Protons, as directly ionizing particles, theoretically exhibit reduced oxygen dependence due to their capacity for direct DNA damage independent of free radical chemistry. Whether statins modulate cellular responses in hypoxic microenvironments exposed to proton beams, and through what mechanisms such modulation might occur, represents uncharted territory requiring systematic investigation before claims of hypoxia-related advantages can be substantiated.

### 4.5. Future Research Agenda

Staged research programs addressing statin–proton synergy must confront several methodological challenges before clinical translation becomes justified. The identification of biomarkers reflecting statin-enhanced radiosensitivity represents a necessary first step, yet current understanding provides limited guidance on which molecular signatures would reliably predict therapeutic benefit. Without validated predictive biomarkers, patient selection for clinical trials remains largely empirical.

LET-dependent experimental investigations assessing statin modulation of RBE, DNA damage, and repair kinetics in prostate cancer models would address fundamental mechanistic uncertainties. These studies must carefully distinguish whether observed effects represent genuine biological synergy or merely additive toxicity. The experimental design should account for temporal dynamics, specifically whether statin effects persist throughout fractionated treatment courses and whether repair inhibition operates consistently across varying LET distributions within proton treatment fields.

The rationale for initiating small prospective trials evaluating concurrent lipophilic statin therapy during proton beam therapy in high-risk prostate cancer depends critically on preclinical data demonstrating a meaningful therapeutic advantage. The decision to proceed with human trials before establishing robust mechanistic foundations risks exposing patients to unproven interventions while consuming limited research resources. The alternative approach of delaying clinical investigation until mechanistic understanding reaches sufficient maturity must be weighed against the possibility of therapeutic benefit for patients with aggressive disease.

Preliminary evidence that supports further investigation would necessitate Phase II/III trials testing whether adjunctive statins improve biochemical control or reduce toxicity compared with proton therapy alone, yet such trials face considerable design challenges. Biochemical control represents a surrogate endpoint whose relationship to overall survival remains imperfect, while toxicity assessment demands extended follow-up periods to capture late effects. The trial design must account for the reality that demonstrating superiority over proton monotherapy, itself representing advanced treatment, demands substantial sample sizes and extended observation periods that challenge feasibility.

## 5. Conclusions

Repurposing statins in oncology offers a transformative opportunity to enhance radiotherapy outcomes, particularly in prostate cancer. The pathways by which statins enhance prostate cancer sensitivity to radiation are of particular interest for creating improvements in local control through various potential synergies with proton therapy. The evidence demonstrates that lipophilic statins consistently show radiosensitization effects in preclinical models, while retrospective clinical evidence and recent large-scale meta-analyses suggest improved biochemical control, particularly in high-risk patients. The large meta-analysis by An et al. demonstrated significant reductions in both prostate cancer-specific mortality and all-cause mortality with statin use. Multiple mechanisms support the rationale for enhanced efficacy through DNA repair inhibition, immune modulation, hypoxia mitigation, and anti-inflammatory effects. High-risk prostate cancer patients appear to derive the greatest benefit from statin–radiotherapy combinations.

However, important limitations must be acknowledged. While recent meta-analyses show overall benefit, individual studies demonstrate considerable heterogeneity in outcomes. All current evidence is derived from photon-based studies, with no direct proton and statin data existing in the literature. Retrospective clinical studies are subject to confounding factors, and optimal statin selection, dosing, and timing remain undefined. Most critically, the proposed statin–PBT synergy is entirely hypothetical and requires experimental validation. Despite these promising aspects, clinical data on statin–proton therapy combinations are entirely absent, necessitating substantial further research. The synergy hypothesized to be uniquely beneficial in prostate cancer due to DNA repair mutation burden, LET sensitivity, RBE enhancement, and dose dependence of outcomes requires rigorous validation through dedicated preclinical studies followed by well-designed prospective clinical trials.

Combining statins with proton therapy represents a promising frontier in radiation oncology that could potentially optimize therapeutic ratios and patient outcomes, making a compelling case for systematic investigation through appropriately designed research programs. However, clinical implementation should await definitive evidence from prospective studies demonstrating safety, efficacy, and optimal treatment protocols.

## Figures and Tables

**Table 1:** Level 1 evidence: summary of preclinical studies investigating statin effects on prostate cancer cells, in vitro studies.

Cell Line	Statin	Radiation	Quantitative Finding	Study
PC-3	Lipophilic statins	No	66.68% reduction in invasion (range 53.93–77.04%). *p* < 0.05	Brown et al. [44]
PC-3	Pravastatin (hydrophilic)	No	No significant effect on invasion	Brown et al. [44]
PC-3, LNCap	Lipophilic statins	Yes	Reduced colony formation, and induced radiosensitization. *p* < 0.05	He et al. [31], Chen et al. [45], Yacoub et al. [46], Oka et al. [26], Hamed et al. [47]
PC-3	Lipophilic statins	Yes	Prolonged life span of radiation-induced ROS. *p* < 0.05	Yu et al. [48]
DU145	Simvastatin	Yes	Dose and time-dependent synergistic effects	Korte et al. [20]

**Table 2:** Level 2 evidence: summary of in vivo studies evaluating statin impact on radiotherapy efficacy and toxicity.

Model	Statin	Radiation	Quantitative Effect	Study
PC-3 xenograft	Lovastatin	Yes	Decreased tumor volume. *p* < 0.05	Hamed et al. [47]
PC-3 xenograft	Simvastatin/Fluvastatin	Yes	Increased tumor oxygenation, Limited impact on tumor growth. *p* < 0.05	d’Hose et al. [49]
Murine lung	Lovastatin	Yes	Reduced lung inflammation and fibrosis. *p* < 0.05	Williams et al. [50]
Balb/c mice	Lovastatin	Yes	Reduced pro-inflammatory responses. *p* < 0.05	Ostrau et al. [43]
Rat model	Pravastatin (Hydrophilic)	Yes	Improved radiation enteropathy. *p* < 0.05	Haydont et al. [51]
C57Bl/6 mice	Pravastatin (Hydrophil)	Yes	Eliminated chronic vascular injury. *p* < 0.05	Ait-Aissa et al. [52]
NOD-SCID mice	Rosuvastatin (Hydrophil)	No	Suppressed xenografted PPC-1 prostate tumors. *p* < 0.05	Wang et al. [53]

**Table 3:** Level 3 evidence: retrospective clinical studies, clinical evidence for improved radiotherapy outcomes with concurrent statin use in prostate cancer.

Sample Size	Radiation	Endpoint	Hazard Ratio	*p*-Value	Notes	Study
N = 1681	86.4 Gy	PSA Relapse-Free Survival	HR 0.69	0.03	Multivariate analysis	Kollmeier et al. [58]
N = 1681	(86.4 Gy)	PSA Relapse-Free Survival	HR 0.52	0.02	Greatest benefit in high-risk patients	Kollmeier et al. [58]
N = 691	EBRT: 72 Gy, Brachytherapy: 144 Gy	Freedom from Biochemical Failure	HR 0.43	0.002	Benefits across all risk groups	Gutt et al. [59]
N = 691	EBRT: 72 Gy, Brachytherapy: 144 Gy	Relapse-Free Survival	HR 0.57	0.005	-	Gutt et al. [59]
N = 567	Radiation not specified	Overall Survival	HR 0.24	<0.001	Hyperlipidemia patients, Post-diagnosis statin use	Li et al. [55]
N = 247	Brachytherapy: 145 Gy, or 110 Gy when combined with EBRT (22–46 Gy)	Biochemical Failure	HR 0.28	<0.01	I-125 brachytherapy, significant benefit	Oh et al. [60]
N = 6537	Radiation not specified	Prostate Cancer Death	HR 0.80	<0.05	Post-diagnosis benefit only	Murtola et al. [57]
N = 3555	EBRT: 74–80 Gy or 45–50 Gy when combined with brachytherapy	Biochemical Recurrence	HR 0.79	NS	No significant benefit or association	Kaulanjan et al. [56]
N = 774	Radiotherapy	Biochemical Recurrence	HR 0.99	NS	No benefit observed	Chao et al. [61]

**Table 4:** Level 4 evidence: systematic review and meta-analysis of statin benefits across cancer types and treatments.

Cancer Type	Radiation	Treatment Context	Endpoint	Hazard Ratio (95% CI)	Number of Studies	Key Finding	Study
Prostate Cancer	Yes	Radiotherapy patients	Biochemical Recurrence	HR 0.78	33 cohort studies	RT-specific benefit	Sun et al. [63]
Prostate Cancer	Mixed	All curative treatments	Biochemical Recurrence	Improved BCRFS	27 studies	High-risk benefit	Yin et al. [65]
Prostate Cancer	Mixed	All treatments	Prostate Cancer-Specific Mortality	HR 0.76	24 studies	Postdiagnostic benefit	An et al. [64]
Prostate Cancer	Yes	Radiotherapy patients	All-Cause Mortality	HR 0.68	24 studies	RT-specific benefit	An et al. [64]
Prostate Cancer	Yes	Radiotherapy patients	Biochemical Recurrence	HR 0.68	35 observational studies	RT-specific benefit	Luo et al. [66]
Prostate Cancer	No	Surgery patients	Biochemical Recurrence	HR 0.97	35 observational studies	No benefit for surgery	Luo et al. [66]
Prostate Cancer	Mixed	Definitive local therapy	Biochemical Recurrence	HR 0.91	8 studies	No significant benefit	Scosyrev et al. [67]
Bladder Cancer	Mixed	All treatments	Overall Survival	No significant benefit	35 observational studies	No benefit	Luo et al. [66]
Renal Cell Carcinoma	Mixed	All treatments	Overall Survival	HR 0.81	35 observational studies	Some benefit	Luo et al. [66]

**Table 5:** Level 5 evidence: randomized controlled trials investigating statin–radiotherapy combinations.

Design	Cancer Type	Sample Size	Radiation	Primary Endpoint	Statin	Status	Study
Phase II/III RCT	Rectal Cancer	N = 316	45–50 Gy Neoadjuvant chemoradiation	Pathological complete response rate	Rosuvastatin 20 mg daily	Ongoing	Patil et al. [68]
Phase II	Prostate Cancer	N = 53	EBRT: 78–89 Gy or 45–46 with brachy therapy. Brachytherapy: 124–145 Gy	Grade ≥ 2 rectal toxicity	Lovastatin 20–80 mg daily	Completed-Primary endpoint not met	Anscher et al. [69]

**Table 6:** Clinical evidence for improved radiotherapy outcomes with concurrent statin use across cancer types.

Cancer Type	Sample Size	Radiation	Endpoint	Hazard/Odds Ratio	*p*-Value	Notes	Study
NSCLC	N = 478	Concurrent chemoradiotherapy	Overall Survival	HR 0.68	0.012	High-intensity statin	Walls et al. [75]
NSCLC	N = 478	Concurrent chemoradiotherapy	Overall Survival	HR 0.70	0.033	Medium-intensity statin	Walls et al. [75]
ESCC	N = 140 (statin users)	Concurrent chemoradiotherapy	Overall Survival	HR 0.65	0.0009	Statin during CCRT	Chen et al. [74]
LSCC	389 (statin users)	Concurrent chemoradiotherapy	Overall Survival	HR 0.60	<0.0001	Statin during CCRT	Yu et al. [73]
Rectal Cancer	N = 407	Neoadjuvant chemoradiation	Pathologic Response	OR 2.25	<0.004	24.3% on statins during treatment	Mace et al. [76]

**Table 7:** Mixed clinical outcomes: special populations and contrasting findings.

Population	Sample Size	Clinical Significance	Study
ADT patients post-RT	N = 1364	Statin benefit in ADT population	Hamilton et al. [77]
Active surveillance	N = 635	No benefit for surveillance outcomes, Limited benefit in low-risk disease	Nyame et al. [78]
IMRT patients	N = 195	Reduced acute rectal toxicity, Protective effect against toxicity	Palumbo et al. [70]
High-risk PCa with RT + ADT	N = 447	No significant benefit, Mixed results in high-risk disease	Cadeddu et al. [79]
High-risk PCa with RT	N = 237	No association with PFS, Recent negative study	Perlow et al. [80]

## Data Availability

No new data were created or analyzed in this study.
